# 23Na-MRI for Breast Cancer Diagnosis and Treatment Monitoring: A Scoping Review

**DOI:** 10.3390/bioengineering12020158

**Published:** 2025-02-06

**Authors:** Taylor Smith, Minh Chau, Jordan Sims, Elio Arruzza

**Affiliations:** 1 Allied Health & Human Performance, University of South Australia, Adelaide, SA 5000, Australia; taylor.smith2104@gmail.com; 2 School of Dentistry and Medical Sciences, Charles Sturt University, Wagga Wagga, NSW 2670, Australia; schau@csu.edu.au; 3 Jones Radiology, Adelaide, SA 5000, Australia; jordan.sims@jonesradiology.com.au

**Keywords:** 23Na-MRI, breast cancer, magnetic resonance imaging, total sodium concentration, sodium, diagnosis, treatment monitoring

## Abstract

(1) Background: Variations in intracellular and extracellular sodium levels have been hypothesized to serve as biomarkers for tumour characterization and therapeutic response. While previous research has explored the feasibility of 23Na-MRI, a comprehensive review of its clinical utility in breast cancer is lacking. This scoping review aims to synthesize existing literature on the potential role of 23Na-MRI in breast cancer diagnosis and treatment monitoring. (2) Methods: This review included English-language studies reporting on quantitative applications of 23Na-MRI in breast cancer. Systematic searches were conducted across PubMed, Emcare, Embase, Scopus, Google Scholar, Cochrane Library, and Medline. (3) Results: Seven primary studies met the inclusion criteria, highlighting the ability of 23Na-MRI to differentiate between malignant and benign breast lesions based on elevated total sodium concentration (TSC) in tumour tissues. 23Na-MRI also showed potential in early prediction of treatment response, with significant reductions in TSC observed in responders. However, the studies varied widely in their protocols, use of phantoms, field strengths, and contrast agent application, limiting inter-study comparability. (4) Conclusion: 23Na-MRI holds promise as a complementary imaging modality for breast cancer diagnosis and treatment monitoring. However, standardization of imaging protocols and technical optimization are essential before it can be translated into clinical practice.

## 1. Introduction

Breast cancer is the most prevalent type of cancer worldwide, contributing to 12.5% of all newly diagnosed cases in 2020 [1]. Growth arises in epithelial cells of either ducts or lobules within the glandular tissue of the breast. This initial growth is benign (in situ), presenting commonly with no symptoms [2]. Without early diagnosis and intervention, in situ (stage 0) cancers can progress and lead to invasion of surrounding breast tissue, nearby lymph nodes (regional metastasis), and potentially other organs within the body (distant metastasis) [2]. Screening and diagnosis are primarily undertaken with mammography which presents a high sensitivity (70–90%), albeit low sensitivity (32–64%) produces increased false positive cases or undetected tumours [3,4]. Moreover, mammography often is unable to distinguish benign from malignant tumours due to the dense tissue of the breast [3,4]. Additional imaging such as ultrasound (US) can increase the sensitivity, however, leads to higher false positive rates [3]. Treatment options can include surgery and various types of therapy (e.g., radiation and chemotherapy) with the primary goal of eliminating neoplastic cells, although response and therapeutic efficiency in vivo prove difficult to progressively monitor [5]. Non-invasive modalities such as PET/CT is able to continually monitor changes in size and metabolic activity of tumours in addition to improved accuracy for detection of recurrence, though this possesses a high radiation dose and increased expense [6].

Magnetic resonance imaging (MRI) is a valuable non-invasive asset in both diagnosis and treatment monitoring of breast cancer, with high sensitivity, specificity, and accuracy [4,7]. In particular, MRI has advantages over mammography in its ability to detect lesions in women with breast implants or dense breast tissue. Clinical trials have also seen visualisation of early indicators of treatment response, including initial lesion patterns, and with dynamic contrast imaging, vascular and cellular changes are trackable [4,7]. MRI diffusion weighted imaging (DWI) is another a specialised quantitative technique that observes microscopic changes in water diffusion across the intra- and extracellular environments, assumed as surrogate markers for metabolic activity [4,7]. Additionally, hydrogen magnetic resonance spectroscopy (1H MRS) provides insight into tumour metabolite concentrations, as increased choline concentration can be characteristic of a malignant lesion [8]. In conjunction with spectral and morphological 1H MRI, recent studies have shown benefits in sodium magnetic resonance imaging (23Na MRI) given its potential in providing metabolic data. This technique requires additional hardware to a clinical scanner, including a dedicated 23Na radiofrequency coil, and involves a radial acquisition that enables ultrashort echo times [8].

Sodium is a vital electrolyte providing a homeostatic environment through osmoregulation to maintain body fluid, blood volume, and pH regulation [9,10]. Concentrations of sodium are, therefore, quite sensitive to metabolic changes of tissues and cellular membranes. The intracellular volume fraction (ISC) is typically larger than the extracellular volume fraction (EVC) within healthy tissue cells [9,10]. Any impairment of energy metabolism or cell membrane integrity will cause an increase in ISC. With the following physiological information, previous research papers have hypothesised that 23Na MRI could provide direct, non-invasive data on ISC and/or EVC due to pathological changes resultant of brain tumours proposed to measure total sodium concentration (TSC) and cell volume fraction (CVF), two quantitative parameters termed “bioscales” [9,10]. TSC is the volume fraction weighted mean of ISC and EVC whereas CVF is the fractional average water content in a normal brain (typically around 0.8mM) [10]. Therefore, [TSC = CVF × ISC + (1-CVF) × EVC] (Poku et al., 2021). By understanding the normal homeostatic TSC, angiogenesis, cell proliferation, and unregulated cell growth (all pathological changes due to cancer) increase the ISC and/or EVC which, therefore, increases the TSC [9,10].

Currently, no scoping reviews have been conducted on the application of Thulborn’s concept of 23Na-MRI to either breast cancer diagnosis or treatment monitoring. Therefore, the objective of this scoping review was to gather a wide breadth of sources to understand the potential role of 23Na-MRI when applied within the diagnosis and treatment monitoring of breast cancer. The results found within this study identify knowledge gaps and collate research undertaken in a key area of advancement towards improved characterisation of breast neoplasms at diagnosis where imaging modalities find suspicious, although inconclusive results. This leads to increased false negatives, late diagnosis, and poor prognosis. Furthermore, we aimed to identify advanced MRI techniques to provide additional biomarkers to contribute to further detailed insights into treatment progress and improved clinical outcomes. This review may serve to identify potential areas of protocol reform and to inform healthcare providers that, as an essential part of a multidisciplinary approach, continued research identifies new approaches for the diagnosis, management, and, therefore, treatment of breast cancer, ultimately leading to improved post-operative recovery and, overall, survival of patients

## 2. Materials and Methods

### 2.1. Literature Search

The full inclusion criteria, search strategy, approaches to study screening, data extraction, and synthesis were stipulated a priori in a protocol and are briefly presented below. This literature search was conducted using the UniSA library to freely access PubMed, Emcare, Embase, Scopus, and Medline. Other databases included Google Scholar and Cochrane Library due to free availability and increased search breadth outside of peer reviewed literature. These were searched in April 2023. Additional studies were gathered from pearling reference lists of full text articles included in the review.

Grey literature was searched using the UniSA Library Catalogue and Google for books, on-peer reviewed sources, government documents, clinical trials, and theses. Most information gathered from grey literature was not included in our study results, though it provided valuable background information regarding the disease and the basic physics behind 23Na-MRI.

### 2.2. Search Strategy

The PCC framework (Table 1) was used to provide a basis for all peer reviewed and non-peer reviewed search strategies. Two researchers (T.S. and E.A.) developed a search strategy within Medline and accomplished MeSH terms reflecting each PCC element including keywords such as “Breast Tumour” and “23Na-MRI” in order to narrow the search. Further keywords were developed for Medline, further narrowing the search results, and the final search strategy was adapted for Emcare and Embase. These keywords were minimised when all other databases as the addition of these would produce minimal or no results. The limitation applied to all strategies included English language only, as this is the common language spoken by all researchers. After removing duplicates within Covidence [11], 640 articles remained for screening (T.S. and E.A.) and full text screening (T.S. and M.C.) with any disagreements resolved by a third party. Keywords used for the systematic search are presented in Appendix A.

### 2.3. Data Extraction

We followed the Cochrane methodology and applied our inclusion and exclusion criteria (refer to Appendix C). The studies that were successful in both stages of screening underwent data extraction by one reviewer (T.S.) within Microsoft Excel [12]. A formatted spreadsheet (refer to Appendix B) was used to organise and outline clear points of data required from each study. Key information extracted included sample size, patient characteristics, protocol (including MRI technical factors, treatment regime and overall study protocol), method of sodium calculation, any relevant quantitative findings (such as TSC, IC, EV, etc.), and qualitative findings (sodium signal intensity, anatomical variances, or clinical responses). Once the studies were successfully extracted, they was independently assessed (E.A.) with any conflicts resolved through discussion.

### 2.4. Study Quality

Of the seven studies included in this review, all were primary in design. Therefore, critical appraisal tools were employed to systematically assess the outcome measures (TSC, ADC, SUV, etc.) through examining factors such as internal validity, relevance, and generalisability to determine the studies methodological quality. For prospective cohort studies, the CASP (Critical Appraisal Skills Programme) Cohort Study Checklist [13] was used as a tool to measure methodological quality. The JBI Cohort study checklist was initially decided for use, however, many of the questions in the checklist related to the quality of the interventional design which required studies to include a control group. Of the three prospective cohort studies, a control group was not employed due to its irrelevance regarding the outcome measures. CASP contains 12 items, including questions about the validity and reliability of results, application of results locally and implications of study. For Controlled Clinical Trials, the JBI Quasi-Experimental study checklist was used. JBI contains 11 items, including questions about the possibility of bias in its design, conduct, and analysis of results. Critical appraisal was performed by two independent reviewers (T.S. and E.A.) both, respectively, assessing all 7 studies individually. Any inconsistencies found were discussed between the appraisers to establish a final decision. The full quality assessment is found in Appendix C.

### 2.5. Synthesis of Results

The studies included were heterogeneous, therefore, data were synthesised using narrative synthesis. Tools and techniques for developing the synthesis included groupings, tabulation, and textual descriptions of studies. The results of the studies were sub-grouped into the phenomena of interest: diagnosis and treatment monitoring. Tabulation assisted in visually representing the results to allow the relationships within and between the studies to be explored. A descriptive paragraph was produced for each individual study to identify its characteristics and their reported findings. This allowed reviewers to compare findings across all included studies. Following the synthesis of results, conclusions were drawn from the body of evidence.

## 3. Results

### 3.1. Study Selection

**Figure 1:**
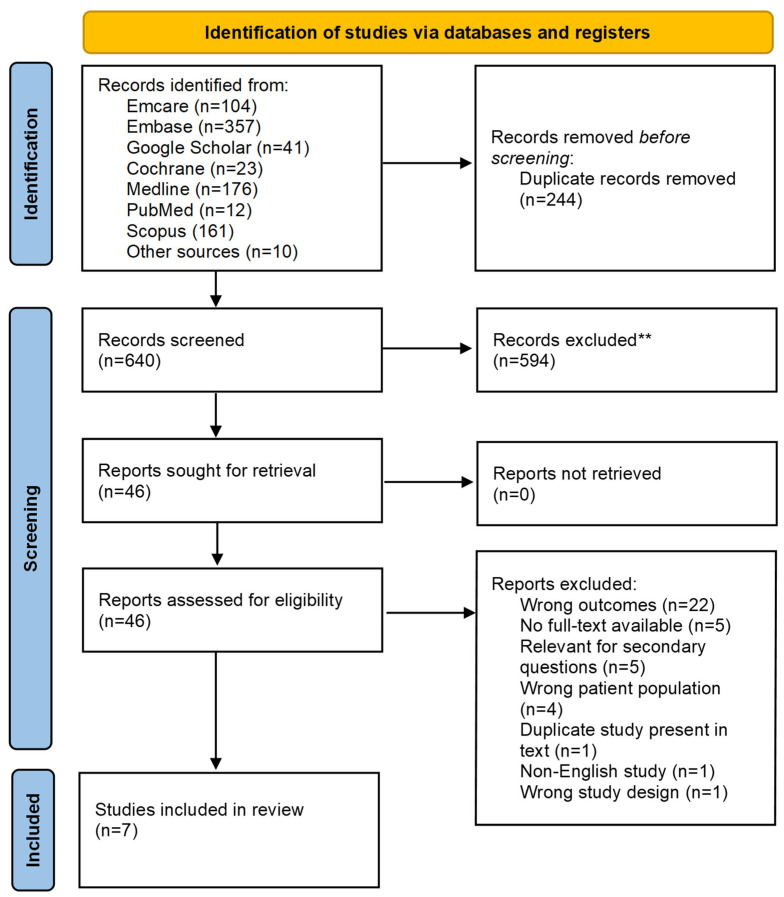
PRISMA flowchart.

There were 884 articles and texts that were yielded from our original search of databases, of which 244 articles and texts were removed as they were duplicates. Of the 640 articles filtered in the initial stage, 594 studies were removed after applying the inclusion and exclusion criteria. Of the 46 that were reviewed in full text, 36 were removed for being irrelevant to our study objectives as they were either using incorrect interventions or incorrect study design. In total, 22 of the articles either did not include 23Na-MRI or did, although, had no information regarding breast cancer. Five articles we could not find access to the full text. Another five articles were excluded as although they contained useful information, they pertained to the secondary questions. Four articles contained participants that were under the age of 18 or had animals as the test subject. One article was removed as it was a thesis summarising other articles included in the full text review. One article was removed as it was written in German which is a language not known amongst the researchers. One article was an abstract only which was an excluded study design. Overall, seven articles were included and form the basis of the scoping review. Figure 1 shows a PRISMA Diagram summarising this process.

### 3.2. Study Characteristics

Table 2 presents a summary of design and patient characteristics of all the included studies. All studies were of primary research, published after 2004. All studies reporting on diagnosis applicability of 23Na-MRI within breast cancer were controlled clinical trials. All studies reporting on treatment monitoring were conducted as prospective cohort studies. The sample size of participants had a small range from 6 to 64, but most studies examined a moderate sample size: 5 studies (70%) included 15–35 participants. All studies included female breast cancer patients over the age of 18, with 4 of 7 studies including additional volunteers or healthy participants as a means of validity and accuracy of sodium measures. There was an almost even distribution of studies reporting on either diagnosis (4 of 7) and treatment monitoring (3 of 7). No studies focused on both diagnosis and treatment monitoring; they had sole focus on either clinical area of interest.

### 3.3. Sodium Quantification Methods

Table 3 presents a summary of MRI characteristics of all the included studies. Sodium quantification methods varied significantly across the included studies, reflecting the lack of standardization in this area. Most studies employed phantoms to calibrate sodium signals, such as ring-shaped phantoms containing 100 mmol/L NaCl solutions [14]. Quantification generally relied on co-registration of proton and sodium images, with MATLAB R2024b scripts frequently used to define regions of interest (ROI) for TSC calculations [14].

However, there were notable variations in phantom designs, reference standards, and calibration protocols. For instance, Zaric et al. (2021) used noise-only scans to adjust for signal-to-noise ratio (SNR) [15], while other studies employed calibration phantoms during each scan session to ensure accuracy [16]. Such inconsistencies highlight the need for standardized approaches to sodium quantification to improve the reproducibility and reliability of findings across different institutions and research settings.

### 3.4. Diagnosis

Ianniello (2021) performed a controlled clinical trial on two BCPs with ten healthy individuals [16]. These breast pathologies were to be triple negative in phenotype. Patient one demonstrated a TSC of 47.0 mM within the tumour cells, in comparison to their healthy glandular tissue demonstrating a TSC of 22.9 mM. Patient two demonstrated a TSC of 39.2 mM within the tumour cells, in comparison to their healthy glandular tissue demonstrating 27.2 mM. These levels of TSC (within tumour tissue) were higher than the ten healthy patients who demonstrated an average TSC of 33.6 mM.

In both patients, they found that CIC was ~130% greater than what is reported in the literature for healthy tissue (10–15 mM) while the ECV was within the physiological values (approximately 0.2). These findings suggest that the increase in TSC mainly originated from an increase in intracellular sodium, which could be due to sodium–potassium pump dysfunction. Additionally, Ianniello found an alteration in the relaxation properties of the tumour due to its molecular environment though this was mitigated by utilising an ultra-short TE sequence (e.g., FLORET). Shortening TE has an adverse effect on T2 weighting, meaning the TSC was underestimated in the lesion with this method. Furthermore, physiological variations of CEC and ECV in the pectoral muscle can affect the quantification of CEC and ECV in the lesion, resulting in a 0.03–15% margin of uncertainty in the calculation of TSC. There was no recorded statistical analysis to determine the differences in concentrations of 23Na. Jacobs (2004) performed a controlled clinical trial with 59 BCPs and 5 healthy volunteers [8]. Patients only needed to be referred for MR evaluation if breast lesions were identified by mammography, ultrasound, or clinical breast examination. There were no individual TSC data published for either the patient or volunteer rather the average values were recorded. The average TSC for BCPs within tumour cells was 47.0mM, with fatty tissue demonstrating a TSC of 20mM and glandular tissue a TSC of 28mM. Volunteers had no numerical data recorded, although it was reported that volunteers had no focal elevations of sodium with breast tissue. Jacobs (2004) used SUN Ultra SPARC60 for their MRI image analysis and statistical analysis using a Student t-test [8]. Further key findings described that sodium imaging assesses different tissue characteristics than either the proton or MRSI exam by reflecting alterations in the cell ionic status or membrane. For example, elevated sodium levels are associated with disruption of the membrane sodium–potassium pump and changes in the water environment within breast tissue.

Ouwerkerk (2007) performed a controlled clinical trial with 22 BCPs. Patients were recruited based on suspicious findings on mammography, ultrasound, or clinical breast examination with a scheduled biopsy [17]. Each patient in the study had their TSC recorded within the lesion, gland, and adipose tissue with 19 patients having malignant tumours and 3 having benign. On average, TSC in malignant lesions was increased by >60% compared to glandular tissue. On average, TSC in benign lesions was about equal to that of the TSC found in non-involved, remote glandular tissue (34 ± 13 mmol/L). For all patients, adipose tissue had less TSC in comparison to lesion and glandular tissue. Malignant lesions demonstrated an increased TSC of 53 ± 16 mmol/L and benign lesions demonstrated an increased TSC of 26 ± 5 mmol/L. Tissue sodium concentration (TSC) was calculated by measuring the mean tissue intensities within the relevant region of interest (ROI) and comparing them to the mean intensity observed in the same location within a concentration reference phantom scan. Sodium concentrations across different tissues within the patient group were analysed using a paired two-tailed t-test. To compare TSC in benign lesions with that in malignant lesions, an independent samples two-tailed t-test was employed. The observed 63% increase in malignant TSC would correspond to either an increase in ICV of 3.2-fold to 38mmol/L > assuming EVF is constant or, almost doubling of the EVF to 34% if ICF is assumed constant or, some combination of both. No acceptable, non-invasive way to directly determine EVF in human breast tissue, but estimate for lesions are available from DCE MRI studies

Zaric 2016 performed a controlled clinical trial with 17 BCPs and 8 healthy patients. Study participants were recruited based on finding suspicious malignancy on mammograms or breast ultrasounds [18]. In total, 20 lesions had a recorded TSC with 15 being malignant and 5 being benign. Malignant tumours presented a TSC mean of 69 +/− 10 mmol/kg with benign tumours recording 47 +/− 8 mmol/kg. In comparison, healthy adipose tissue recorded 18 +/− 3 mmol/kg, and glandular tissue recorded 35 +/− 3 mmol/kg. 23Na demonstrated good differentiation between malignant and benign breast lesions with an inverse correlation between ADC. TSC assessed with 23Na is sensitive to intrinsic changes linked to tumour malignancy as TSC is sensitive to changes in cell volume fraction as a result of cellular death, swelling, and proliferation, as well as changes in intracellular sodium content due to impaired energy metabolism or other metabolic changes that affect sodium exchange across the cell membrane. Additionally, quantification in necrotic tissue or any other nonviable part of the tumour can bias measured values of TSC and ADC. Zaric (2016) did not record statistical analysis to determine the differences in concentrations of 23Na [18].

#### 3.4.1. Treatment Regime

The treatment regimens varied across studies depending on the chemotherapy protocols used. In Jacobs (2010), patients received an anthracycline-based regimen consisting of intravenous doxorubicin (60 mg/m^2^) and cyclophosphamide (600 mg/m^2^), followed by an intravenous injection of either paclitaxel (175 mg/m^2^) or docetaxel (100 mg/m^2^) [14]. These were administered every 21 days for four cycles, spanning approximately 12 weeks. In a subsequent study, Jacobs (2011) employed a different regimen where patients were administered docetaxel (100 mg/m^2^) every 14 days for 4 cycles, with an option for additional courses of doxorubicin and cyclophosphamide based on clinical response [19]. In contrast, Zaric (2021) utilized a neoadjuvant chemotherapy (NACT) protocol comprising epirubicin (90 mg/m^2^) and cyclophosphamide (600 mg/m^2^) along with docetaxel (100 mg/m^2^), repeated every three weeks for four cycles. Patients with HER2-positive tumours additionally received targeted therapies, including trastuzumab and pertuzumab, as part of their treatment regimen [15].

#### 3.4.2. Treatment Monitoring and Outcome

Jacobs (2010) scanned their participants three times during their prospective cohort study [14]. Initial baseline measurements were acquired before the participant’s first treatment cycle, second measurements after their first cycle of treatment (within 14 days), and the last measurements after treatment (prior to surgery). In total, eighteen participants had stage two or three breast cancer and were candidates for pre-operative systematic therapy (PST). Responders to treatment saw a mean baseline TSC of 66 +/− 18 mmol/L decrease to a mean of 48.4 +/− 8 mmol/L. This translates to a decrease in TSC by a range of 17–26%. Non-responders to treatment saw a mean baseline TSC of 52 +/− 8 mmol/L increase to a mean of 56 +/−2 mmol/L. This translates to an increase in TSC by 9%. Significant decreases in choline SNR and TSC were observed in responders after the first cycle of treatment (had higher AUCs). Stable TSCs with smaller decreases in choline SNRs were noted in non-responders after the first cycle, and had lower AUCs. Large decreases in TSC were seen in patients with TN or HER2+ who were responders. Little or no changes in TSC occurred in patients with TN or HER2+ who were non-responders. Most PST is cytotoxic in nature and will induce changes in the tumour cellular membrane, with the hope of disrupting the intracellular and extracellular function of tumour cell, therefore, affecting intracellular TSC will lead to decreased TSC. The study involved calculating sodium levels by using a ring-shaped phantom containing a 100 mmol/L NaCl solution as a reference, with 1H MR images co-registered to 23Na images via MATLAB. These co-registered images helped guide the placement of regions of interest to determine tissue sodium concentration (TSC), signal-to-noise ratio (SNR), and contrast-to-noise ratio. Statistical analysis was conducted to evaluate changes in 23Na concentrations before and after treatment using paired t-tests and analysis of variance, and these results were compared with final pathological outcomes. Clinical response was assessed in the breast and regional lymph nodes through examinations conducted at study enrollment, after each cycle of pre-surgical therapy (PST) and before surgery, with tumor response evaluated by palpation. Histologic analyses were performed by a breast pathologist as part of routine care, and the pathological response was classified as complete, partial, or no response based on the presence and extent of invasive cancer cells. Patients achieving complete or partial responses were categorized as responders, while all others were considered non-responders [14].

Jacobs 2011 scanned their participants two times during their prospective cohort study. Initial baseline measurements were acquired before the first treatment cycle and final measurements were acquired after administration of the participant’s first treatment cycle (within 8 days) [19]. For five out of six patients, Jacobs 2011 saw a mean TSC decrease of 21% within breast tissue of the six participants that had stage two or three breast cancer and were candidates for pre-operative systematic therapy (PST) [19]. TSC calculations were performed for all six individuals, including before and after treatment. Mean baseline TSC was recorded at 5.2mM with mean TSC after the first cycle of treatment recorded at 42.2mM. In patient six, they exhibited a mean increase of 3% after the first cycle (56.2 → 58.1mM). Overall, patients one to five exhibited a mean tumour volume decrease of 42% in responders and 35% in non-responders. Significant decreases in TSC and SUV metrics were observed in responders, consistent with histological changes (decrease in proliferation index (Ki-67) in responders and increase in non-responders). Tissue sodium concentration (TSC) in breast tissues was calculated by co-registering proton images using in-house MATLAB scripts and drawing regions of interest (ROI) on both proton and 23Na images. The TSC was determined in both lesion and normal breast tissue as previously described. Clinical response was assessed by a board-certified breast medical oncologist through examinations conducted at study enrollment, before each pre-surgical therapy (PST) session, and prior to surgery, including evaluations of the breast and relevant lymphatic regions. Histological tissue classification was used to determine the pathologic response, categorizing it as complete (pCR), partial (pPR), or no response (pNR) based on the extent of invasive cancer cells. Statistical analysis was conducted using ANOVA and unpaired t-tests to evaluate differences in MRI-defined volumes and 23Na concentrations before and after treatment.

Zaric 2021 scanned their participants three times during their prospective cohort study [14]. Initial baseline measurements were acquired before the participant’s first treatment cycle, second measurements after their first cycle of treatment (within 14 days), and the last measurements after their second treatment cycle (within 14 days). Of the 15 participants they consecutively presented with findings for primary breast cancer, and who were scheduled to undergo NACT. The mean tumour TSC at baseline was 70.6 mmol/L ± 6.0 mmol/L. For participants with a pCR, TSC reduction after the first and second cycles was 12.0% (8.3 mmol/L ± 69.4 mmol/L) and 28.5% (19.8 mmol/L ± 69.4 mmol/L), respectively. In participants without a pCR, TSC reduction after the first cycle was 4.7% (3.4 mmol/L ± 71.7 mmol/L), and after the second cycle, TSC was reduced by 5.4% (3.9 mmol/L ± 71.7 mmol/L). The greatest reduction of TSC was found after the first cycle within patients who achieved a pCR and in those with triple negative phenotype. The results obtained from interaction tests between treatment time and participants’ responses showed that the difference in TSC between participants with and without a pCR was not the same after the first and second cycles. TSC differed in participants with and without a pCR and between the first and second chemotherapy cycles. Reduction can better differentiate between participants with and without a pCR after the first chemotherapy cycle than 2D tumour size reduction. TSC between the first and second chemotherapy cycles was not the same for participants with and without a pCR. A two-way mixed model analysis of variance was conducted to determine if the changes in tumour size or tissue sodium concentration (TSC) over time differed between participants with a pathologic complete response (pCR) and those without. The difference in TSC values of healthy glandular tissue, adjusted using correction factors from phantom and in vivo data, was evaluated using a t-test to assess how well tumour size or TSC reduction could distinguish between participants with and without a pCR. Post-hoc paired t-tests were performed to compare the differences in TSC and tumour size between baseline and the first chemotherapy cycle, as well as baseline and the second chemotherapy cycle, for both groups of participants [14].

**Table 3:** MRI characteristics.

Author	RF Coil	Number of RF Channels	MRI Manufacturer	Tesla	Protocol
Ianniello 2021 [16]	Dual-tuned transmit-receive breast coil (proton and sodium)	Sodium = 8 Proton = 2	Siemens Healthineers	7 T	^1^H T1 fat-suppressed 3D-VIBE for anatomical reference 4 × 3D-VIBE datasets with different TE’s to calculate water fraction with IDEAL method Acquire sodium images with FLORET Proton T1 maps (MP2RAGE) acquired pre and 10min post Gadavist injection (0.1 mL/Kg) DCE 1H T1 3D-VIBE FS for contrast perfusion
Jacobs 2004 [8]	Custom solenoid sodium coil designed to fit within phased array breast coil	N/A	General Electronic Healthcare	1.5 T	Sagittal T2 FS fast spin echo T1 FSPGR 3D T1 FSPGR pre and post 0.1 mmol/kg Omniscan (acquisition started immediately after injection completion) PRESS—272 ms TE (with CHESS and STIR for water/lipid suppression) Double gradient echo images to calculate B0 strength and assess patient movement during PRESS 23Na-MRI using TPI
Jacobs 2010 [14]	Custom solenoid sodium coil designed to fit within phased array breast coil	N/A	General Electronic Healthcare	1.5 T	Sagittal T2 FS fast spin echo T1 FSPGR 3D T1 FSPGR pre and post 0.1 mmol/kg Omniscan (acquisition started immediately after injection completion) PRESS—280 ms TE (with CHESS and STIR for water/lipid suppression) 23Na-MRI using a projection imaging sequence
Jacobs 2011 [19]	Custom sodium coil designed to fit within phased array breast coil	N/A	General Electronic Healthcare	1.5 T	Sagittal T2 FS fast spin echo T1 FSPGR 3D T1 FSPGR pre and post 0.1 mmol/kg Omniscan (acquisition started immediately after injection completion) 23Na MRI using a modified projection imaging sequence
Ouwerkerk 2007 [17]	Custom solenoid sodium coil designed to fit within phased array breast coil	N/A	General Electronic Healthcare	1.5 T	Sagittal T2 FS fast spin echo T1 FSPGR 3D T1 FSPGR pre and post 0.1 mmol/kg Omniscan (acquisition started immediately after injection completion) 23Na MRI using TPI
Zaric 2016 [15]	1H imaging: dual-tuned breast coil (proton and phosphorus) 23Na imaging: dual-tuned breast coil (proton and sodium)	Sodium Receive Only = 6 Sodium Transmit and Receive = 2 Proton Receive Only = 2	Siemens Healthineers	7 T	Transverse 23Na acquisition-weighted stack of spirals sequence Transverse DWI—EPI T1 DCE—time resolved angiography with stochastic trajectories (0.1 mmol/Kg Dotarem)
Zaric 2021 [18]	Dual-tuned breast coil (proton and sodium)	Sodium = 14 Proton = 2	Siemens Healthineers	7 T	23Na MRI via density-adapted, three-dimensional radial projection reconstruction pulse sequence (with additional noise only scans) 1H non contrast T1 DIXON (fat + water images)

Footnotes: TPI refers to twisted projection imaging, a specialized MRI sequence that enhances spatial resolution and minimizes acquisition time by twisting data projections around the k-space center. UTE, or ultrashort echo time, is an MRI sequence designed to capture signals from tissues with short T2 relaxation times, improving the visualization of sodium ions. DCE-MRI (dynamic contrast-enhanced MRI) is a technique that tracks tissue perfusion and vascular properties using contrast agents, providing valuable insights into tumor vascularization and treatment response.

## 4. Discussion

This scoping review presents the most up-to-date literature on the application of 23Na-MRI for breast cancer diagnosis and treatment monitoring, summarizing key findings from various studies and identifying gaps and challenges in its clinical implementation. Although 23Na-MRI has shown promise in differentiating malignant from benign breast lesions and in monitoring treatment response, several limitations and technical considerations must be addressed before it can be widely adopted in clinical practice.

Incorporating 23Na-MRI into routine diagnostic pathways faces significant challenges. The existing imaging modalities, such as proton MRI, contrast-enhanced mammography, ultrasound, and PET/CT, are already well-established and offer high sensitivity and specificity in detecting breast cancer. While 23Na-MRI provides unique insights into TSC and cellular microenvironment changes, the additional time required for sodium imaging, often ranging from 20 to 40 min, significantly extends the overall scan duration. This could negatively impact patient comfort, particularly for those already anxious or stressed due to the nature of the examination. Studies have not sufficiently addressed these patient-centered considerations, highlighting a critical gap that must be filled to justify its routine clinical use. Further, this review found that the standardization of protocols, coil selection, and sodium concentration quantification methods varied widely among the studies. For example, Ianniello (2021) and Zaric (2016; 2021) used dual-tuned coils for sodium and proton imaging, whereas other studies employed custom sodium coils fitted within existing breast coils [14,15,18]. The dual-tuned coils offered more detailed signal detection but also introduced complexities in isolating intracellular sodium concentrations. Additionally, the use of contrast agents was inconsistent; while some studies incorporated Omniscan or Dotarem, others omitted contrast altogether, raising questions about the impact of contrast on TSC sensitivity and specificity. Given that contrast-enhanced imaging may also elevate risks such as NSF and gadolinium retention, particularly with repeated scans, this variability complicates the establishment of a standard imaging protocol.

A major challenge in the integration of 23Na-MRI is the optimization of imaging protocols to minimize user and scanner biases. All studies, excluding Jacobs (2004), used phantoms to map signal references and fiducial markers for optimal registration with 1H images [8]. Phantoms and calibration methods varied, affecting the reliability of quantitative TSC data. For instance, Ianniello (2021) utilized calibration phantoms for each scan session, while Zaric (2016) and Ouwerkerk (2007) employed different phantom setups for testing signal-to-noise ratio (SNR) and linearity, thereby complicating inter-study comparisons [15,16,18]. Establishing a standard methodology for TSC quantification is crucial to reduce variability and improve the reproducibility of results across different MRI systems and settings.

Another important consideration is the impact of magnetic field strength on 23Na-MRI measurements. While higher field strengths, such as 7T, offer increased SNR and potentially greater spatial resolution, they are more susceptible to artifacts, B0 and B1 inhomogeneities, and chemical shift effects. This was evident in studies such as Zaric (2016; 2021) and Ianniello (2021), which reported greater impairments in image quality at 7T compared to 1.5T systems [15,16]. Although 7T MRI may provide more detailed data, the practical benefits in clinical settings remain questionable given the reduced image quality and increased hardware requirements. Additionally, the choice of MRI manufacturer, whether Siemens or GE, could influence artefact management and image quality, further complicating standardization efforts.

A significant challenge in the clinical adoption of 23Na-MRI is the lack of consistency in results across institutions. Variations in imaging protocols, calibration methods, scanner hardware, and software configurations contribute to discrepancies in sodium quantification. For example, studies such as Jacobs et al. (2004) and Zaric et al. (2016) used different calibration phantoms and quantification methods, which affected the reproducibility of TSC measurements [8,18]. Additionally, differences in field strength (e.g., 1.5 T vs. 7 T) and radiofrequency coil designs (custom vs. dual-tuned coils) further exacerbate these inconsistencies. Insights from sodium MRI applications in other cancers, such as brain tumors, reveal similar challenges, where standardization of imaging protocols significantly improved inter-institutional comparability [10].

Moreover, 23Na-MRI is currently more suited for research and treatment monitoring than routine diagnostic use. Although studies such as Jacobs (2010) and Jacobs (2011) demonstrated that TSC could serve as an early biomarker of treatment response, several challenges remain [14,19]. Extended scan times, repeated administration of contrast agents, and the associated risks, such as patient discomfort, heat stress, peripheral nerve stimulation, and safety considerations related to SAR regulations for implants and devices, limit its feasibility for widespread clinical adoption. These safety considerations, combined with logistical challenges in integrating sodium imaging protocols into standard workflows, highlight the need for standardized protocols, advanced sequence optimization, and multicenter trials. Furthermore, the repeated use of contrast agents increases the risk of adverse effects, such as gadolinium retention and nephrogenic systemic fibrosis [20]. Future research should explore strategies to reduce scan times, minimize the use of contrast agents, and incorporate patient feedback to improve the overall experience of 23Na-MRI.

Sodium MRI has shown potential beyond breast cancer, particularly in lung and prostate cancers, where TSC has been used as a biomarker for tumour characterization and therapeutic response [21,22,23]. These studies highlight the technique’s sensitivity to metabolic and cellular changes, although inter-study variability remains a barrier to broader clinical adoption. Leveraging lessons from these applications may offer strategies to enhance standardization and reproducibility in breast cancer imaging.

Given the complexities of implementing 23Na-MRI in a clinical setting, future research should focus on developing shorter scan protocols, improving coil technology, and refining quantification methods. For example, advancements in radial acquisition techniques and UTE sequences could help reduce scan duration without compromising image quality. Additionally, the development of standardized phantom designs and calibration procedures would facilitate multicenter trials and improve the comparability of results. Establishing guidelines for protocol optimization, safety considerations, and patient management will be essential to determine whether the benefits of 23Na-MRI outweigh its drawbacks. The primary strength of this review is its rigorous methodology, which included a systematic search and appraisal of studies using well-established frameworks such as the PRISMA guidelines. By incorporating a diverse range of study designs (controlled clinical trials, prospective cohorts, and quasi-experimental studies), this review offers a broad perspective on the feasibility and potential impact of 23Na-MRI. Furthermore, the detailed comparison of MRI protocols, coil configurations, and sodium quantification techniques provides valuable insights for researchers seeking to standardize 23Na-MRI applications and improve inter-study reproducibility. However, several limitations must be considered when interpreting the findings. Firstly, the heterogeneity in study protocols, magnetic field strengths, and data acquisition techniques complicates direct comparisons between studies and limits the generalizability of the results. The generalizability of the findings is also further restricted by the regional and demographic limitations of existing studies, which predominantly involve small cohorts from specific geographic areas. The reviewed studies varied significantly in their use of phantoms, patient characteristics, and sodium quantification methods, leading to potential biases and inconsistencies in reported outcomes. For instance, while some studies employed dual-tuned coils for simultaneous hydrogen and sodium imaging, others used custom sodium coils, resulting in differing SNR and spatial resolutions.

## 5. Conclusions

This scoping review provides a comprehensive evaluation of the role of 23Na-MRI in breast cancer diagnosis and treatment monitoring, synthesizing evidence from a range of primary studies. Although 23Na-MRI has shown potential to enhance diagnostic specificity and serve as an early biomarker for treatment response, its clinical implementation is currently limited by several factors, including extended scan times, technical complexities, and the need for additional hardware. The variation in protocols, coil designs, and sodium quantification methods across the reviewed studies further underscores the need for standardization to ensure reproducibility and reliability. Moreover, the requirement for specialized equipment and software, coupled with safety concerns related to prolonged scan times and repetitive use of contrast agents, restricts its applicability to specialized research settings. While 23Na-MRI provides unique metabolic insights into breast tumour physiology that could complement conventional imaging modalities, its integration into routine clinical workflows remains challenging.

## Figures and Tables

**Table 1:** PCC Framework (2023).

*Population*	Female breast cancer patients >18 years (malignant and benign)
*Concept*	^23^Na magnetic resonance imaging (relating to diagnosis and treatment monitoring)
*Context*	N/A * (Research published in English only)

**Table 2:** Study design and participant characteristics [14,15,16,17,18,19].

Author	Country	Study Duration	Population	Number of Participants	Study Design	Clinical Area of Interest	Study Protocol
Ianniello 2011 [16]	USA	Nil	TNBC (histology not specified)	BCP = 2 Healthy Patients = 3 Volunteers = 10	Controlled Clinical Trial	Diagnosis	^23^Na-MRI (TSC, ECV, ICV, C_IC_) ^1^H-MRI (as anatomical reference)
Jacobs 2004 [8]	USA	Nil	Malignant and benign lesions (histology not specified)	BCP = 59 Healthy Volunteers = 5	Controlled Clinical Trial	Diagnosis	^23^Na-MRI (TSC) ^1^H-MRI (Angular Separation) MRSI (Choline SNR)
Jacobs 2010 [14]	USA	Nil	Ductal, lobular, and mixed	BCP = 18	Prospective Cohort	Treatment Monitoring	^23^Na-MRI (TSC, SNR, Contrast-to-Noise Ratio) ^1^H-MRI (RECIST and Volume Analysis with DCE) MRSI (Choline SNR)
Jacobs 2011 [19]	USA	Nil	IDC and ILC	BCP = 6	Prospective Cohort	Treatment Monitoring	^23^Na-MRI (TSC) ^1^H-MRI (Volume Analysis with DCE) PET/CT (SUV using ^18^FDG) Core Biopsy (Proliferation Index/Ki-67)
Ouwerkerk 2007 [17]	USA	Nil	IDC, DCIS, ILC, fibrocystic benign, fibroadenoma	BCP = 22	Controlled Clinical Trial	Diagnosis	^23^Na-MRI (TSC, SNR) ^1^H-MRI (Anatomical Reference with DCE)
Zaric 2016 [15]	Austria	September 2013–November 2014	IDC, ILC, and DCIS (malignant) Papilloma and fibroadenoma (benign)	BCP = 17 Healthy Volunteers = 8	Controlled Clinical Trial	Diagnosis	^23^Na-MRI (TSC) ^1^H-MRI (Lesion Size with DCE) DWI (ADC) Core Biopsy (Proliferation Index/Ki-67)
Zaric 2021 [18]	Austria	June 2018–July 2019	IDC	BCP = 15 Healthy Volunteers = 6	Prospective Cohort	Treatment Monitoring	^23^Na-MRI (TSC) ^1^H-MRI (Lesion Size with DIXON) Core Biopsy (Proliferation Index/Ki-67)

## Appendix A

**Table A1:** Medline database search strategy (Performed on 1 April 2023).

#	Searches	Results
1	Breast/	44,237
2	Breast Neoplasms/	324,517
3	(“breast tumo?r” or “benign breast tumo?r” or “malignant breast tumo?r” or “Ca breast” or “breast Ca” or “Cx breast” or “Breast Cx” or “Breast Carcinom?” or “Breast Neoplasm*” or “Breast Lesion*”).mp. [mp = title, book title, abstract, original title, name of substance word, subject heading word, floating sub-heading word, keyword heading word, organism supplementary concept word, protocol supplementary concept word, rare disease supplementary concept word, unique identifier, synonyms]	347,737
4	1 or 2 or 3	365,584
5	(“Sodium 23” or “Na23” or “23Na” or “23 Sodium” or “Total Sodium Concentration” or “Sodium Concentration” or “Na-MRI” or “Sodium-MRI” or “Sodium-MR” or “Sodium Magnetic Resonance” or “Na Magnetic resonance” or “23Na-MR*” or “23NaMR*” or “23 Na MR*” or “Sodium MRI” or “MR Spectroscopy” or “Sodium Quantification” or “23Na Sodium MR” or “23Na Sodium MRI” or “multinuclear Na-MRI” or “Multiparametric Na-MRI” or “multiparametric sodium magnetic resonance imaging” or “multiparametric sodium magnetic resonance” or “MRI spectroscopy” or “multinuclear 23Na imaging” or “tissue sodium concentration” or “sodium accumulation”).mp. [mp = title, book title, abstract, original title, name of substance word, subject heading word, floating sub-heading word, keyword heading word, organism supplementary concept word, protocol supplementary concept word, rare disease supplementary concept word, unique identifier, synonyms]	12,113
6	4 and 5	174

**Table A2:** Emcare database search strategy (Performed on 1 April 2023).

#	Searches	Results
1	breast/ or breast cancer/ or breast carcinoma/	90,612
2	(“breast tumo?r” or “benign breast tumo?r” or “malignant breast tumo?r” or “Ca breast” or “breast Ca” or “Cx breast” or “Breast Cx” or “Breast Carcinom?” or “Breast Neoplasm*” or “Breast Lesion*”).mp. [mp = title, abstract, heading word, drug trade name, original title, device manufacturer, drug manufacturer, device trade name, keyword heading word]	24,805
3	1 or 2	99,368
4	(“Sodium 23” or “Na23” or “23Na” or “23 Sodium” or “Total Sodium Concentration” or “Sodium Concentration” or “Na-MRI” or “Sodium-MRI” or “Sodium-MR” or “Sodium Magnetic Resonance” or “Na Magnetic resonance” or “23Na-MR*” or “23NaMR*” or “23 Na MR*” or “Sodium MRI” or “MR Spectroscopy” or “Sodium Quantification” or “23Na Sodium MR” or “23Na Sodium MRI” or “multinuclear MRI” or “Multiparametric MRI” or “multiparametric magnetic resonance imaging” or “multiparametric magnetic resonance” or “MRI spectroscopy” or “multinuclear 23Na imaging” or “tissue sodium concentration” or “sodium accumulation”).mp. [mp = title, abstract, heading word, drug trade name, original title, device manufacturer, drug manufacturer, device trade name, keyword heading word]	5,355
5	3 and 4	142

**Table A3:** Embase database search strategy (Performed on 1 April 2023).

#	Searches	Results
1	breast/ or breast cancer/ or breast carcinoma/	557,333
2	(“breast tumo?r” or “benign breast tumo?r” or “malignant breast tumo?r” or “Ca breast” or “breast Ca” or “Cx breast” or “Breast Cx” or “Breast Carcinom?” or “Breast Neoplasm*” or “Breast Lesion*”).mp. [mp = title, abstract, heading word, drug trade name, original title, device manufacturer, drug manufacturer, device trade name, keyword heading word, floating subheading word, candidate term word]	221,023
3	1 or 2	658,290
4	(“Sodium 23” or “Na23” or “23Na” or “23 Sodium” or “Total Sodium Concentration” or “Sodium Concentration” or “Na-MRI” or “Sodium-MRI” or “Sodium-MR” or “Sodium Magnetic Resonance” or “Na Magnetic resonance” or “23Na-MR*” or “23NaMR*” or “23 Na MR*” or “Sodium MRI” or “MR Spectroscopy” or “Sodium Quantification” or “23Na Sodium MR” or “23Na Sodium MRI” or “multinuclear MRI” or “Multiparametric MRI” or “multiparametric magnetic resonance imaging” or “multiparametric magnetic resonance” or “MRI spectroscopy” or “multinuclear 23Na imaging” or “tissue sodium concentration” or “sodium accumulation”).mp. [mp = title, abstract, heading word, drug trade name, original title, device manufacturer, drug manufacturer, device trade name, keyword heading word, floating subheading word, candidate term word]	26,454
5	3 and 4	577

## Appendix B

**Table A4:** Data information extracted from each study.

Source Type	Data to be Extracted
**Peer-reviewed**	Publication details (author, publication date, journal) Study Design Study Setting (hospital, research facility, private clinic, rural, urban) Study Purpose (diagnosis, treatment monitoring, feasibility) Sample Size Participant Age (mean, median and range) Participant Characteristics (healthy or suspicious finding, tumour type, tumour size, histologic type, phenotype, lesion location) Area Scanned (unilateral or bilateral) Participant Eligibility Criteria MRI Characteristics (Tesla, commercial name, RF coil used ▯ combined ^1^H and ^23^Na or independent ^23^Na coil) Protocol Used (weighting, sequences, contrast, fat saturation) Conflicts of Interests Image Pre/Post Processing Method (manual or software) Image Analysis/Evaluation Method (software, analytical, statistical, human) ○ Blinded or non-blinded, Comparison to reference imaging, classification system (BIRADS, RECIST) Study Limitations Method of Quantitative Calculation ○ In vivo quantification used (phantom, additional participants) Quantitative Outcomes: TSC (Total Sodium Concentration (mmol/L)) ECV (Extracellular Volume Fraction) ISC (Intracellular Volume Fraction) C_IC_ (Intracellular Sodium Concentration) STQ (Sodium Triple-Quantum Signal) SNR (Signal-to-Noise Ratio) Choline MRS (Magnetic Resonance Spectrum) Qualitative Outcomes: Comments on image quality Radiographic appearance (criterion used or appearances observed) ^23^Na MRI correlation with other imaging methods (e.g., DWI, ADC) Method of observed clinical response (progression of images, histology, tumour size, etc.) Key findings related to ^23^Na-MRI Any additional information relevant (cost-effectiveness, accessibility, radiographer availability, time taken to image, availability of test, experience of patients or clinicians)
**Non-peer reviewed**	Credibility (source location, author credibility, qualifications, and previous publications, area of specialist), date of source (still valid and reflects current standards, pieces of evidence, new information), publication details (date, source for any data taken), reference list (source reliable and up-to-date), organisation (author affiliations, conflicts of interests, reputable organisation), consistency with other sources (both peer and non-peer reviewed, specificity of information), purpose of source (who, what, why, when was this source created for), language employed (formal, casual, broad, emotions and impressions evoked), domain type (if from website, owning organisation).

## Appendix C

**Table A5:** JBI quasi-experimental critical appraisal checklist.

	Q1	Q2	Q3	Q4	Q5	Q6	Q7	Q8	Q9
**Ianniello 2011**									
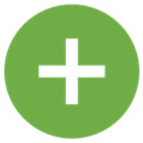
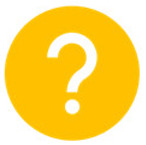
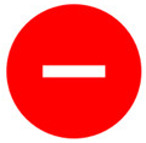
**Jacobs 2004**									
**Ouwerkerk 2007**									
**Zaric** **2016**									

***Question 1:*** Is it clear in the study what is the cause and what is the effect?

***Question 2:*** Were the participants included in any comparisons similar?

***Question 3:*** Were the participants in any comparisons receiving similar treatment/care, other than the exposure or intervention of interest?

***Question 4:*** Was there a control group?

***Question 5:*** Were there multiple measurements of the outcome both pre- and post-intervention/exposure?

***Question 6:*** Was follow-up complete and if not, were differences between groups in terms of their follow-up adequately described and analysed?

***Question 7:*** Were the outcomes of participants included in any comparisons measured in the same way?

***Question 8:*** Were outcomes measured in a reliable way?

***Question 9:*** Was appropriate statistical analysis used?

**Table A6:** CASP cohort study checklist.

	Q1	Q2	Q3	Q4	Q5	Q6	Q7	Q8	Q9	Q10
**Jacobs 2010**										
**Jacobs 2011**										
**Zaric 2021**										

***Question 1:*** Did the study address a clearly focused issue?

***Question 2:*** Was the cohort recruited in an acceptable way?

***Question 3:*** Was the exposure accurately measured to minimise bias?

***Question 4:*** Was the outcome accurately measured to minimise bias?

***Question 5:*** Have the authors identified all-important co-founding factors in the design and/or analysis?

***Question 6:*** Was the follow-up of subjects complete and long enough?

***Question 7:*** Do you believe the results?

***Question 8:*** Can the results be applied to the local population?

***Question 9:*** Do the results of this study fit with other available evidence?

***Question 10:*** What are the implications of this study for practice?

**Table A7:** JBI cohort study critical appraisal checklist.

	Q1	Q2	Q3	Q4	Q5	Q6	Q7	Q8	Q9	Q10	Q11
**Jacobs 2010**											
**Jacobs 2011**											
**Zaric 2021**											

***Question 1:*** Were the two groups similar and recruited from the same population?

***Question 2:*** Were the exposures measured similarly to assign people to both exposed and unexposed groups?

***Question 3:*** Was the exposure measured in a valid and reliable way?

***Question 4:*** Were confounding factors identified?

***Question 5:*** Were strategies to deal with co-founding factors stated?

***Question 6:*** Were the groups/participants free of the outcome at the start of the study (or from the moment of exposure)?

***Question 7:*** Were the outcomes measured in a valid and reliable way?

***Question 8:*** Was the follow-up time reported and sufficient to be long enough for outcomes to occur?

***Question 9:*** Was the follow-up complete, and if not, were the reasons to loss of follow-up described and explored?

***Question 10:*** Were strategies to address incomplete follow-up utilised?

***Question 11:*** Was appropriate statistical analysis used?
